# High-throughput mammographic-density measurement: a tool for risk prediction of breast cancer

**DOI:** 10.1186/bcr3238

**Published:** 2012-07-30

**Authors:** Jingmei Li, Laszlo Szekely, Louise Eriksson, Boel Heddson, Ann Sundbom, Kamila Czene, Per Hall, Keith Humphreys

**Affiliations:** 1 Department of Medical Epidemiology and Biostatistics, Karolinska Institutet, Box 281, Stockholm 17177, Sweden; 2 Human Genetics Lab, Genome Institute of Singapore, 60 Biopolis Street, 02-01, Singapore 138672, Singapore; 3 Department of Microbiology, Tumor and Cell Biology, Karolinska Institutet, Box 280 Stockholm 17177, Sweden; 4 Sweden Karolinska Institutet Visualization Core Facility (KIVIF), Karolinska Institutet, Stockholm 171 77, Sweden; 5 Mammography Skåne, Unilabs AB, Helsingborg 252 23, Sweden; 6 Breast Center/Mammography Unit, Södersjukhuset AB, Stockholm 118 83, Sweden

## Abstract

**Introduction:**

Mammographic density (MD) is a strong, independent risk factor for breast cancer, but measuring MD is time consuming and reader dependent. Objective MD measurement in a high-throughput fashion would enable its wider use as a biomarker for breast cancer. We use a public domain image-processing software for the fully automated analysis of MD and penalized regression to construct a measure that mimics a well-established semiautomated measure (Cumulus). We also describe measures that incorporate additional features of mammographic images for improving the risk associations of MD and breast cancer risk.

**Methods:**

We randomly partitioned our dataset into a training set for model building (733 cases, 748 controls) and a test set for model assessment (765 cases, 747 controls). The Pearson product-moment correlation coefficient (*r*) was used to compare the MD measurements by Cumulus and our automated measure, which mimics Cumulus. The likelihood ratio test was used to validate the performance of logistic regression models for breast cancer risk, which included our measure capturing additional information in mammographic images.

**Results:**

We observed a high correlation between the Cumulus measure and our measure mimicking Cumulus (*r *= 0.884; 95% CI, 0.872 to 0.894) in an external test set. Adding a variable, which includes extra information to percentage density, significantly improved the fit of the logistic regression model of breast cancer risk (*P *= 0.0002).

**Conclusions:**

Our results demonstrate the potential to facilitate the integration of mammographic density measurements into large-scale research studies and subsequently into clinical practice.

## Introduction

Extensive mammographic density (MD) is a strong risk factor for breast cancer. MD refers to the different radiologic patterns of dense and nondense tissue in the breast. Radiologically dense tissue (for example, connective and epithelial tissue) appears light on a mammogram [1]. Nondense tissue is made up mostly of fat, is radiologically lucent, and appears dark on a mammogram. Women with dense tissue in more than 75% of the breast have been consistently reported to be at a four- to sixfold higher risk of developing the disease than are women of similar age with little or no dense tissue [2-4]. A substantial fraction of breast cancers can be attributed to this risk factor. One third of all breast cancers have been found to be diagnosed in women with more than 50% density [5].

MD can be evaluated and reported by radiologists on the basis of visual analysis of mammograms. Examples of quantitative and qualitative classification methods based on the visual characterization of mammographic parenchymal patterns include BIRADS, Wolfe [6], and Tabar [7]. *C*omputer*-*assisted methods are also used to assess MD. The interactive thresholding technique introduced by Byng *et al*. [8], Cumulus, has been validated as being predictive of breast cancer risk in many large epidemiologic studies, and has thus gained acceptance as the gold standard for acquiring quantitative MD reads. Screen-film mammograms must be digitized before using Cumulus. An operator selects the threshold grey levels that identify specific regions of the breast. Two thresholds are chosen by the operator: one to outline the edge of the breast, and the other to distinguish dense breast tissue from nondense breast tissue. Percentage density (PD) is calculated by an algorithm that identifies the number of pixels in each category.

MD is not yet an integral part of predicting the risk of breast cancer at screening and has limited influence in the clinical decision-making process for breast cancer-preventive interventions. A key challenge in the incorporation of MD data in research studies or clinical practice is that the assessment of MD by using the described methods, when performed on a large scale, is heavily restricted because of time and cost. The second challenge is that these methods are to some extent dependent on a subjective interpretation by the reader, some more so than others. A robust automatic method that measures MD, developed to work in a high-throughput setting, would thus be of great benefit to both single assessments of MD and longitudinal studies assessing risk of breast cancer with respect to MD change in large-scale screening programs.

We present a fully automated method of assessing MD quantitatively from digitized analogous film mammograms by using ImageJ [9], a public domain, Java-based image-processing program developed at the National Institutes of Health. This method was developed with two intentions. The first intention was to duplicate findings of the established semiautomated method (Cumulus), and the second, to explore the value of additional features of mammographic images for explaining breast cancer risk. We estimated breast cancer risks associated with MD measurements acquired by using both Cumulus and our method mimicking Cumulus, and compared the discriminatory power between the two measurements in a large population-based case-control study consisting of 1,498 breast cancer cases and 1,495 healthy controls. Coupled with further modifications designed to improve the risk associations of mammographic density and breast cancer risk, we also illustrated that mammograms hold information over and above PD that can improve prediction of breast cancer outcome.

## Materials and methods

### Main study population

This study is an extension of a breast cancer case-control study carried out among Swedish residents born in Sweden and aged 50 to 74 years, between October 1, 1993, and March 31, 1995 [10,11]. Information on breast cancer risk factors was collected from self-reported questionnaires. The study was approved by the ethical review board at Karolinska Institutet, and by the five ethical review boards in other regions in Sweden. All participants provided informed consent.

Postmenopausal women with incident primary invasive breast cancer were identified via the six Swedish Regional Cancer Registries. The 3,979 women with a diagnosis of invasive breast cancer were identified, and 84% (3,345) of these women participated in the study. The primary reasons for nonparticipation were patient's refusal or doctor's refusal because of the patient's poor health.

Controls were frequency matched by the expected age distribution (5-year intervals) among cases and identified through the Swedish National Population Register holding data on national registration number, name, address, and place of birth of all Swedish residents. The response rate among controls was 82% (3,455 of 4,188).

### Retrieval and digitization of mammograms

We sought to retrieve all mammograms for the eligible women in the initial cohort of the main study population by using the Swedish national registration numbers (described in Ludvigsson *et al*. [12]). We could thereby obtain addresses for participants from 1975 to 1995 through the civil registry. During 2006 through 2008, we visited all mammography screening units and radiology departments conducting screening mammography throughout Sweden. We collected all available mammograms for the study participants, up to and including 1995 for controls and until date of diagnosis for cases, and obtained 29,077 film mammograms for 3,859 study subjects.

Film mammograms of the mediolateral oblique (MLO) view were digitized by using an Array 2905HD Laser Film Digitizer, which covers a range of 0 to 4.7 optical densities. The density resolution was set at 12-bit dynamic range. For participants in this study with multiple mammograms, the most recent mammogram was used; for cases, this was the mammogram before diagnosis. The mammogram contralateral to the tumor was chosen for cases. If this image was missing, the examination before the most recent examination was selected. For controls, we randomized side and used the same procedure as for cases. Women with bilateral breast cancer were excluded.

Cases lacking information on tumor side or lacking films of the contralateral breast were excluded, as were subjects with previous reduction mammoplasty, and subjects who only had mammograms of very poor quality. There were 3,593 participants with eligible film mammograms (1,784 cases and 1,809 controls).

### Assessment of mammographic density

#### Current gold standard method: Cumulus

Mammographic density was measured by using the Cumulus software, a computer-assisted technique developed at the University of Toronto, Ontario, Canada [8]. For each image, a trained observer (LE) set the appropriate gray-scale threshold levels defining the edge of the breast and distinguishing dense from nondense tissue. The software calculated the total number of pixels within the entire region of interest and within the region identified as dense. The percentage density was then calculated from these values (dense area/total breast area). The images measured in this study were part of a larger study in which approximately 4,000 images were measured. Images for breast cancer cases were measured together with almost the same number of images for healthy women, and the reader was blinded to case-control status. A random 10% of the images were included as replicates to assess the intraobserver reliability, which was high, with an R^2^-squared of 0.95. In addition, LE regularly calibrated herself against a training set of mammograms measured by Professor Boyd, an expert on, and one of the developers of, Cumulus [3].

#### Novel automated thresholding method

To process automatically the digitized film mammograms and to measure PD, we used ImageJ [9], a public domain Java image-processing program.

### Preprocessing to remove patient identification tags and standardize images

Patient-identification tags were first automatically removed (cropped) by ImageJ from the images. Further preprocessing of the images was required to extract the breast region from the rest of the image. Background of the image was subtracted by superimposing a "mask" derived by applying grayscale erosion and gaussian Blur filters, followed by implementing the Kittler and Illingworth Minimum Error thresholding [13], implemented in the Auto Threshold (v1.10) ImageJ plugin [14]. Although preprocessing was satisfactory for most images, traces of unremoved tags were present in a small subset of mammograms. As the general patient-identification tag placement of film mammograms differed between centers, manual inspection of the preprocessed images was carried out to ensure proper removal of artefacts. Wherever possible, remaining artefacts were manually corrected. In total, 2,993 mammograms corresponding to 1,498 cases and 1,495 controls were retained for further analysis.

### Automated image thresholding

**Figure 1:**
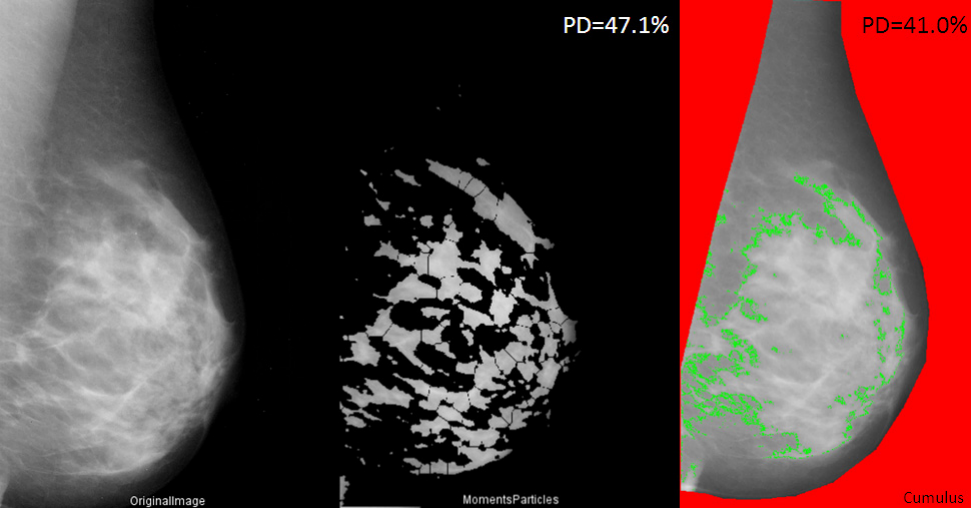
**Examples of processed images**. An example of a digitized mammogram before and after thresholding and application of the watershed algorithm (one image, one particular thresholding algorithm, Moments), and the same image thresholded by using Cumulus.

Having identified the breast region of the image, we further applied automated thresholding methods to separate the areas of "dense" breast tissue ("regions of interest") from the remaining area of the breast. In total, 15 thresholding methods, which vary according to the type of pixel-intensity information they exploit (such as histogram shape, clustering, entropy) were applied to the preprocessed image, and the same preprocessed image after subtracting background by using a rolling-ball algorithm and further filtering/de-noising of the image. Areas corresponding to "dense" tissues were subdivided into smaller objects by using the watershed algorithm. Figure 1 shows an example of a digitized mammogram before and after thresholding and application of the watershed algorithm (one image, one particular thresholding algorithm, Moments), and the same image thresholded by using Cumulus. A more elaborate example illustrating images thresholded by all different algorithms is provided in Additional file 1, Figure S1.

**Figure 2:**
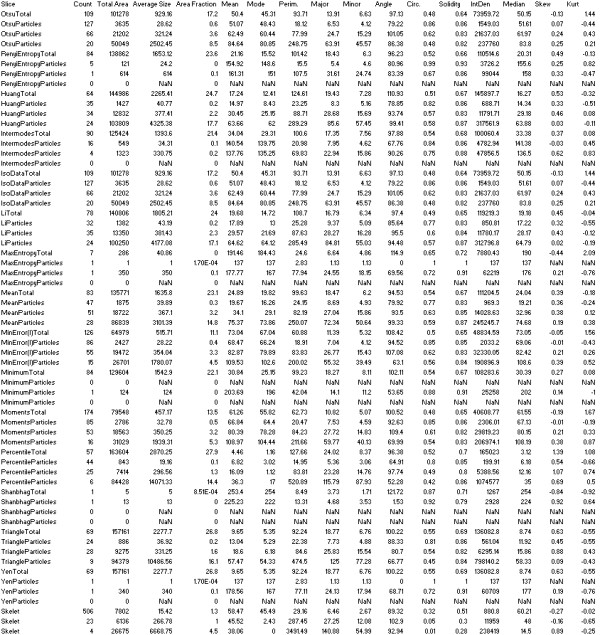
**ImageJ output**. An example of the output file from ImageJ, which includes 1,008 variables.

The Analyze command in ImageJ was then used to count and measure objects in the thresholded images (for groups of objects divided into four size categories: 5+ in the case of the former preprocessed images; 1 to 100, 101 to 1,000, and 1,001+ pixels, in the case of the latter images that underwent background subtraction and watershedding). A variety of measurements were obtained for the breast as a whole, as well as for the "objects" of dense tissue, under each thresholding method (details given in Table 1). We also used the Analyze command in ImageJ, after applying the "find edges" filter in ImageJ to identify sharp changes in intensity, and binary thinning to find the centerlines of objects in the image (in place of thresholding). For each image, 1,008 measurements were obtained as output from ImageJ. An example of the output file from ImageJ is shown in Figure 2.

**Table 1:** Types of measurements made

Area measurements	
Count	Numbers of particles
TotalArea	Area of selection in square pixels
AverageSize	Average size of each particle (TotalArea divided by count)
Area Fraction	The percentage of pixels in the image or selection that have been thresholded

**Intensity measurements**	

Mean gray value	Average gray value within the selection. This is the sum of the gray values of all the pixels in the selection divided by the number of pixels
Modal gray value	Most frequently occurring gray value within the selection. Corresponds to the highest peak in the histogram
Median	The median value of the pixels in the image or selection

**Shape descriptors**	

Circularity	4π (area/perimeter^2^). A value of 1.0 indicates a perfect circle. As the value approaches 0.0, it indicates an increasingly elongated polygon. Values may not be valid for very small particles
Solidity	Area/convex area

**Others**	

Integrated density	The sum of the values of the pixels in the image or selection. This is equivalent to the product of Area and Mean Gray Value
Skewness	The third-order moment about the mean
Kurtosis	The fourth-order moment about the mean
Perimeter	The length of the outside boundary of the selection
Fit ellipse	Fit an ellipse to the selection. Uses the headings Major, Minor, and Angle. Major and Minor are the primary and secondary axis of the best-fitting ellipse. Angle is the angle between the primary axis and a line parallel to the × axis of the image

The Analyze command in ImageJ counts and measures objects in thresholded images. It works by scanning the selection until it finds the edge of an object. It then outlines the object by using the wand tool, measures it by using the Measure command, fills it to make it invisible, and then resumes scanning until it reaches the end of the image or selection.

Not all of the measurements/variables produced by ImageJ were informative (for example, a large number of images lacked objects of a particular size, under particular thresholding procedures). Analysis was limited to 772 variables with less than 200 NaN ("not a number") values. All remaining NaN values in the 772 variables were converted to zero in subsequent analyses.

### Statistical analysis

#### Descriptive variables

The two-sample Student *t *test was used to compare the means of continuous variables. Because of the nonnormal distributions of MD measures, we used the nonparametric Wilcoxon test to compare the distribution of percentage density and absolute dense area. Distributions of categoric variables were compared by using the χ^2 ^test. All tests were two-sided.

#### Machine-learning method to estimate MD measures

To build and assess a PD estimation model, we randomly partitioned the dataset, consisting of information on 2,993 women, into two parts: a training set for model building (733 breast cancer cases and 748 healthy controls), and a test set for model assessment (765 cases and 747 controls).

Principal component analysis was used to carry out feature selection. Instead of directly using the 772 nonindependent "raw" (ImageJ) variables, for building a model of PD, we substituted, in their place, 123 principal components (PCs). The weights (of the raw variables) used by each PC were calculated from a principal component analysis (PCA) of the training set. These 123 PCs captured 90% of the total variance of the original 772 variables (in the training set). The Scree plot, showing the fraction of total variance, as explained or represented by each PC, is displayed in Additional file 2, Figure S2. Weights for each of the original variables (loadings) for each PC are listed in Additional file 3, Table S1.

Our first aim was to select a model for Cumulus PD, as a function of the PCs. As other researchers have done [15], we worked with the square-root transformation of PD to ensure approximate normality. Model selection was based on penalized estimation of a linear model by using the lasso (l1) penalty [16,17]. The method minimizes the residual sum of squares subject to a constraint on the sum of the absolute values of the regression coefficients. The purpose of this shrinkage is to prevent overfitting the data because of either collinearity of the covariates or high-dimensionality. The penalized package in R [18] was used to find optimal values of the shrinkage tuning parameter (lambda) by using repeated tenfold likelihood cross-validation. The data in the training set was repeatedly broken into 10 sets of n/10 women. During each run, nine subsets of data were used to fit the models, and the remaining "validation" set was used to compute the likelihood value for model selection. Tenfold cross-validation was repeated 100 times to obtain a mean lambda for the model. To obtain the final model for PD, the linear model using the lasso penalty, based on the optimal value of lambda, was fitted to the full training set. Our "ImageJ PD" measure is derived by summing the products of the regression coefficients of this model with the corresponding PC values of that image (and also including the intercept). The test set was then used for "external" assessment of the predictive accuracy of the "trained" ImageJ PD measure.

The same procedure may be applied to get a trained estimate of other MD measures by ImageJ, such as total breast area, absolute dense area, or absolute nondense area.

#### Comparison of MD measured by Cumulus and ImageJ

To test for an association between Cumulus PD and ImageJ PD, the Pearson product-moment correlation coefficient (*r*) was estimated. The Bland-Altman plot was used to assess the agreement between the two methods of measurement.

Percentage density is often divided into six categories [3], but because of small numbers of subjects in some categories of mammographic density, we created a new low category (<5%) and combined the upper three categories (25% to 50%, 50% to 75%, and >75%). The odds ratios (ORs) and corresponding 95% confidence intervals (CIs) for risk of breast cancer associated with different categories of mammographic density were estimated by using unconditional logistic regression.

The power to discriminate breast cancer case-control status by using estimates of PD (ImageJ and Cumulus) was evaluated by calculating the area under the curve (AUC) of the receiver operating characteristic (ROC) curve. The pROC package in R was used to calculate AUCs along with their standard errors and 95% confidence intervals. The DeLong test [19] was used to compare the areas under two different ROC curves.

#### Evaluation of extra information from mammograms that is associated with breast cancer over and beyond PD

In addition to using ImageJ to mimic the Cumulus measure of PD, we performed a systematic evaluation of the information in the ImageJ variables in terms of their ability to predict breast cancer risk. We fitted logistic regression models with the lasso penalty in the training set (consisting of 733 cases and 748 controls) by using a similar procedure to that described earlier for the linear model for PD. The same 123 PCs were included in our analysis, and repeated 10-fold cross validation was again used to obtain the optimal value of the tuning parameter. We "trained" three models:

1. 123 PCs as covariates; all regression coefficients included in the penalty term.

2. PD + 123 PCs as covariates; coefficients for the 123 PCs included in the penalty term, but not the coefficient for PD.

3. PD + 123 PCs as covariates; all coefficients (123 PCs + PD) included in the penalty.

Based on these three models, we formed three "scores" for each image, derived by summing the products of the nonzero regression coefficients of the PCs with the corresponding PC values of that image. We refer to these scores as score 1, score 2, and score 3 (according to these three models). The test set was then used for "external" assessment of the predictive ability of the "trained" ImageJ scores; we fitted logistic regression models with breast cancer status as outcome variable, with different combinations of the scores and Cumulus or ImageJ PD as covariates.

R (version 2.13.0) [20] was used for data management, statistical analyses and graphics. All reported tests are two-sided.

## Results

A summary of descriptive statistics of breast cancer risk factors for the subjects included in this study, presented by breast cancer case status, is shown in Table 2. Significant univariate associations with breast cancer status, in the same direction as previously reported in the literature, were seen for percentage density (*P *< 0.001), absolute dense area (*P *< 0.001), age at diagnosis or reference date (*P *< 0.001), age at mammogram (*P *< 0.001), age at menopause (*P *= 0.001), alcohol consumption (*P *= 0.008), parity/age at first birth (*P *< 0.001), hormone-replacement therapy (*P *< 0.001), family history of breast cancer (*P *< 0.001), and benign breast disease (*P *< 0.001). A near-significant association was observed for age at menarche (*P *= 0.084). Body mass index (BMI) (*P *= 0.801) was not significantly associated with breast cancer case status in this subset of the main population case-control study.

**Table 2:** Summary characteristics of study population by breast cancer case status

Characteristic	Cases (*n *= 1,498)	Controls (*n *= 1,495)	*P*
**Median**			
Percentage density (%)	16.9	11.0	<0.001
Absolute dense area (cm^2^)	24.3	16.9	<0.001
			
**Mean (SD)**			
Age at diagnosis or reference date (y)	61.2 (7.1)	62.8 (6.9)	<0.001
Age at mammogram (y)	60.6 (7.2)	62.8 (6.7)	<0.001
Age at menarche (y)	13.5 (1.4)	13.6 (1.4)	0.084
Age at menopause (y)	50.6 (3.8)	50.0 (3.9)	0.001
BMI at diagnosis or reference date (kg/m^2^)	25.1 (3.6)	25.2 (3.9)	0.801
Alcohol consumption (g/day)	2.8 (5.0)	2.3 (4.2)	0.008
Percentage density (%)	20.7 (16.1)	15.7 (14.5)	<0.001
Absolute dense area (cm^2^)	30.2 (23.7)	23.3 (21.5)	<0.001
			
**Frequency, number (%)**			
Categoric percentage density (%)			<0.001
<10	477 (31.8)	680 (45.5)	
10-24	538 (35.9)	506 (33.8)	
25-49	384 (25.6)	250 (16.7)	
50-74	96 (6.4)	57 (3.8)	
≥75	3 (0.2)	2 (0.1)	
Categoric absolute dense area (cm^2^)			<0.001
<10	288 (19.2)	475 (31.8)	
10-24	471 (31.4)	509 (34.0)	
25-49	460 (30.7)	347 (23.2)	
50-74	211 (14.1)	114 (7.6)	
75-99	47 (3.1)	39 (2.6)	
≥100	21 (1.4)	11(0.7)	
Parity and age at first birth			<0.001
Nulliparous	190 (12.7)	148 (9.9)	
1-3 children, age at first birth <25 y	556 (37.1)	555 (37.1)	
1-3 children, age at first birth 25-29 y	420 (28.0)	399 (26.7)	
1-3 children, age at first birth ≥30 y	223 (14.9)	212 (14.2)	
≥4 children, age at first birth <25 y	29 (1.9)	31 (2.1)	
≥4 children, age at first birth ≥25 y	78 (5.2)	150 (10.0)	
Hormone replacement therapy			<0.001
Never used hormones	722 (48.2)	844 (56.5)	
Ever used hormones	774 (51.7)	649 (43.4)	
Unknown status of hormone use	2 (0.1)	2 (0.1)	
Family history of breast cancer (ever)	207 (13.8)	119 (8.0)	<0.001
Benign breast disease (ever)	351 (23.4)	141 (9.4)	<0.001

Descriptive statistics for the study population according to training and test sets are given in Additional file 4, Table S2. For all variables examined, we observed no significant difference in summary statistic between the two data sets.

**Figure 3:**
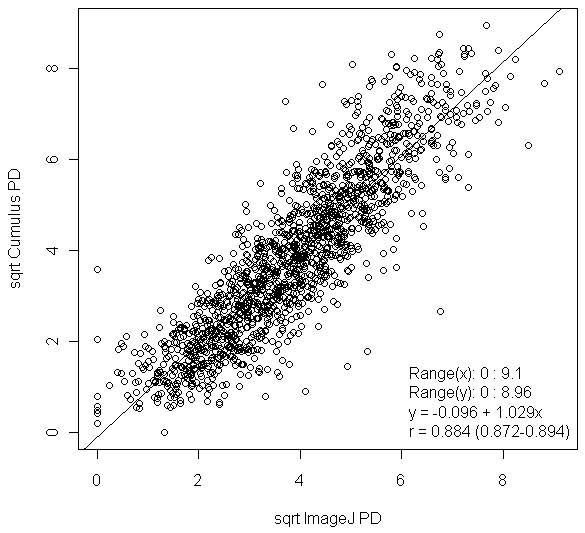
**Scatterplot of Cumulus PD and ImageJ PD**. Sqrt, square-root transformed; r, Pearson product-moment correlation coefficient; x, x-axis; y, y-axis.

**Figure 4:**
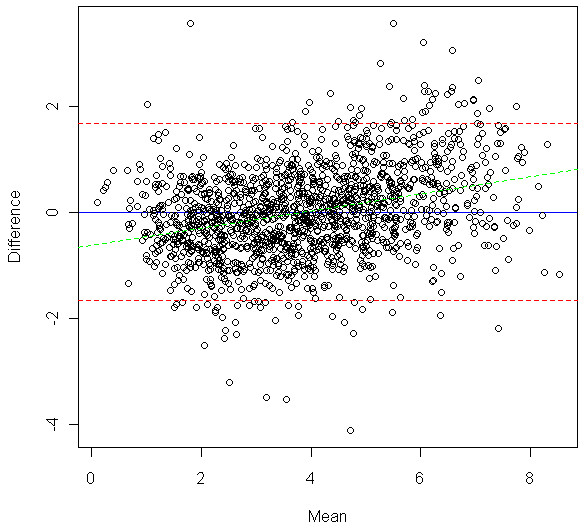
**Bland-Altman plot of mean of Cumulus PD and ImageJ PD versus the difference between the two measurements**. Blue solid line, Mean difference (0.019). Red dotted lines, Lower and upper limits of agreement (-1.66 and 1.69, respectively). Green dotted line, Line of best fit (slope = 0.161; intercept = -0.610).

We externally evaluated the performance of our ImageJ PD measure, derived by using the training set, by assessing the correlation between Cumulus PD and ImageJ PD in the test set. We observed a high correlation (*r *= 0.884; 95% CI, 0.872 to 0.894; Figure 3) between Cumulus PD and ImageJ PD measurements. The corresponding correlation in the training data was naturally higher (*r *= 0.902; 95% CI, 0.892 to 0.911). From fitting a linear regression model (test set), we found that a 1% increase in the value of ImageJ PD was associated with a 1.029% increase in the value of Cumulus PD. The range of square-root transformed PD values for ImageJ (0 to 9.10) was similar to that of Cumulus (0 to 8.96). Bland-Altman analysis for Cumulus PD and ImageJ PD, based on the 1,512 samples in the test set, showed good agreement (*r *= 0.311; 95% CI, 0.265 to 0.356; slope = 0.161; intercept = -0.610; Figure 4). The mean difference in PD between the two methods was 0.019 (95% CI, -1.66 to 1.69).

**Figure 5:**
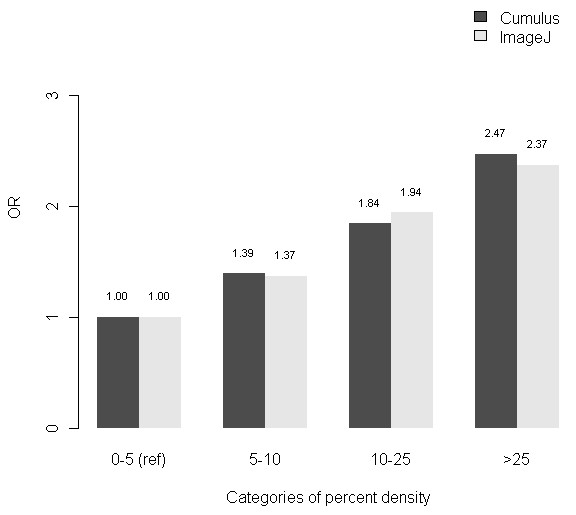
**Risk profiles of Cumulus PD and ImageJ PD**. OR, Odds ratio from unconditional logistic regression of breast cancer risk among cases and controls.

**Figure 6:**
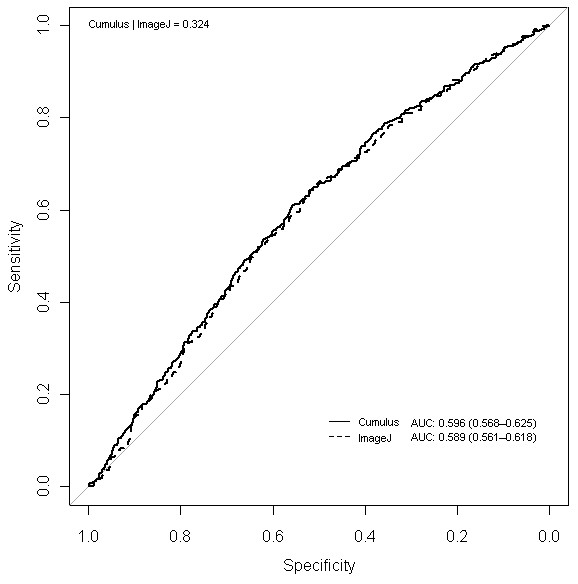
**Discriminatory powers of Cumulus and ImageJ for predicting breast cancer risk, as measured by area under curve (AUC)**. Legend on the top-left corner summarizes *P *values for the Delong test between two receiver operating characteristic (ROC) curves. Legend on the bottom-right corner summarizes the AUC for each model with corresponding 95% confidence intervals.

The breast cancer risk profiles (OR and corresponding 95% CI) were similar for both Cumulus PD and ImageJ PD (Figure 5). The AUCs for Cumulus PD (0.596; 95% CI, 0.568 to 0.625) and ImageJ PD (0.589; 95% CI, 0.561 to 0.618) were not significantly different from one another (Delong *P *= 0.324; Figure 6).

Having assessed the ability of our ImageJ PD measure to mimic Cumulus PD, we next turned to whether ImageJ might hold extra information (not captured by Cumulus) for discriminating cases and controls. We selected three models based on the training set. Three scores were constructed as linear combinations of 28, 3, and 37 PCs from fitting penalized regression models (see Statistical analysis), respectively. As expected, score 3 included considerably more PCs than score 2 and was more strongly associated with PD (*r *= 0.370; 95% CI, 0.326 to 0.413, as compared with *r *= 0.094; 95% CI, 0.044 to 0.144). Not including PD as a penalized covariate during the process of forming score 2, meant that score 2 was "forced" to be independent of PD, whereas score 3 was constructed based on a model that penalized PD along with the PCs. Additional file 5 shows the nonzero coefficients of the three penalized regression models. Table 3 summarizes the goodness of fit of eight logistic regression models for the test set. Score 1, which was derived from 123 PCs (using the training set), was strongly associated with breast cancer risk in the test set, and the residual deviance of this model was also lower than the models with Cumulus and ImageJ PD only. Models of breast cancer risk that included either score 2 or score 3, together with PD, improved the goodness of fit of models that included only PD (for both Cumulus PD and ImageJ PD). The improvement in fit due to including score 3, in addition to PD, was strongly (*P *= 0.0002 for both Cumulus and ImageJ) significant, indicating that information is contained in mammographic images, not captured by PD, that is important for discriminating between cases and controls. The extra information captured by ImageJ is independent of PD in predicting risk. We also note that the fully automated ImageJ PD + score 3 model has a lower Akaike information criterion (AIC) [21] than the semiautomated Cumulus PD model (2,053.3 compared with 2,059.6).

**Table 3:** Goodness of fit of eight logistic regression models fitted to the test set

Model	Null/Residual deviance	P1	P2
Null	2,095.9	-	-
Cumulus PD	2,057.6	6.3 × 10^-10^	-
ImageJ PD	2,062.8	8.8 × 10^-9^	-
Score 1	2,052.5	4.5 × 10^-11^	-
Cumulus PD + score 2	2,053.5	6.4 × 10^-10^	0.0424
Cumulus PD + score 3	2,043.8	4.9 × 10^-10^	0.0002
ImageJ PD + score 2	2,058.7	8.4 × 10^-9^	0.0427
ImageJ PD + score 3	2,049.3	7.7 × 10^-11^	0.0002

Scores for individual images in the test set were derived by summing the products of the nonzero regression coefficients (estimated by using the training set) by the corresponding PC values of that individual image: (1) 123 principal components (PCs) as covariates; all regression coefficients included in the penalty term; (2) percentage density (PD) + 123 PCs as covariates; coefficients for the 123 PCs included in the penalty term, but not the coefficient for PD; and (3) PD + 123 PCs as covariates; all coefficients (123 PCs + PD) included in the penalty. P1, based on Likelihood Ratio Test, comparison with null model. P2, based on Likelihood Ratio Test, comparison with model including Cumulus or ImageJ PD.

## Discussion

We developed an automated thresholding method for obtaining quantitative measurements of MD that compares favorably with the established semiautomatic computer-assisted Cumulus method in predicting risk of breast cancer. The algorithm is based on an established Java-based image-analyses program, ImageJ. Furthermore, we showed evidence that additional features in a mammogram captured by ImageJ, summed into a collective score, represent a significant and independent marker of breast cancer risk.

Other researchers have developed automated approaches to measure MD. For example, Heine *et al*. [22] described an automated breast-density method, based on the analysis of wavelet-filtered images, which directly measures PD as the ratio of segmented dense tissue to the total area of the breast. The authors compared their continuous percentage MD measurements with those acquired by Cumulus. Kallenberg *et al*. [23] describe a method that, like our approach, extracts a number of features from the pixels in mammographic images and uses these to train (and validate) a measure of PD against a "ground truth" (Cumulus PD). Our MD measurement was associated with a correlation (*r *= 0.875; 0.863 to 0.887), which was similar to that of Kallenberg *et al*. [23] (*r *= 0.895), and substantially higher than that of Heine *et al*. [22] (*r *= 0.70). In our study, the odds ratios associated with breast cancer risk were also similar between PD measured by Cumulus and ImageJ, suggesting that PD measured by ImageJ is as good as PD measured by Cumulus in indicating the likely development of breast cancers. Kallenberg *et al*. [23] included only healthy women in their study and were thus unable to make a similar comparison.

It could be said that the AUCs of both ImageJ and Cumulus PD are relatively low (in the range of 0.589 to 0.596) compared with what has been reported before, for example, for the parenchymal pattern-based BIRADS density measure (AUC = 0.658) [24]. However, the fairly low AUC that we observed may be connected to the characteristics of our study population (postmenopausal women). The AUC values can vary to a large extent across different populations; for the original Gail model, for instance, the reported AUC values have ranged between 0.54 and 0.74 (0.54 in a cohort of 70-year-old and older U.S. women [25] and 0.74 in a study of UK women aged 21 to 73 from a UK family-history clinic [26]. Moreover, the Cumulus method, on which our PD measure is trained, has been reported to have better intraobserver reliability than BIRADS [27], and the proposed method, by being reader independent, has merit in terms of intra- and interreader reliability.

In the present study, we provided evidence that our approach captures additional information in mammographic images, in addition to PD, which improves the ability to discriminate between breast cancer disease status, when compared with using PD alone (*P *= 0.0002). ImageJ might be capturing information not related to PD, for example, features related to mammographic texture, in the mammograms. PCs with nonzero coefficients in each score (listed in Additional file 5) are in turn linear combinations of the "original" variables (see Additional file 3), so in principle, it is possible to interpret the scores. In practice, however, it is difficult to provide clear interpretations. PC axes will generally not coincide exactly with any of the original variables, often making interpretations for the PCs very challenging. Nevertheless, we observe that PCs with nonzero coefficients in score 3 are generally less area and intensity measurements, and more shape descriptors or variables describing fitted ellipses, in contrast to PCs for ImageJ PD, which are more closely related to area and intensity variables. We have, however, presented the strongest evidence so far that mammographic images contain additional information to Cumulus PD, which improves the ability to discriminate between breast cancer disease statuses, but further work is needed to clarify exactly what information our score captures.

Although the relation between mammographic breast density and breast cancer risk has been clearly demonstrated, studies have also shown that a potential independent relation exists between mammographic parenchymal texture and the risk of breast cancer [28]. Nielsen *et al*. [28] describe an algorithm that extracts textural information from all pixels of segmented breast images, which is "trained" to recognize texture relating to breast cancer status of the women. Their texture-resemblance marker significantly improved the ability to discriminate disease status in a sample of 245 breast cancer cases and 250 healthy controls, independent of a computer-based PD score resembling Cumulus. It appears that predictive accuracy for breast cancer is increased by adding a "qualitative" measure, akin to previous methods described by Wolfe [6] and Tabar [7], to quantitative estimates of MD.

We based our method on an established and dependable image-processing program developed at the National Institutes of Health (NIH) that is freely available. ImageJ can automatically open, process, and analyze a digitized mammogram in less than 12 seconds, offering a huge advantage over time-consuming measurements using Cumulus, which typically takes a reader between 2 and 5 minutes to achieve the same result. The software runs on Java and is thus not based on any specific platform and is inexpensive in terms of expertise needed to run the macros for processing mammograms. Its open-source design makes ImageJ more easily evolvable and correctable than many proprietary packages, allowing the fine-tuning of parameters for nonstandard mammograms or potentially, with further development, non-film mammograms. As the PD estimates are derived from a machine-learning-based method, the data can be easily retrained to output other measures, such as absolute dense and nondense areas. An additional strength includes our large population-based breast cancer case-control study, which allows us to apply and validate ImageJ PD alongside Cumulus PD in the estimation of breast cancer risk.

A robust, automated thresholding method would shorten the time and cost needed to acquire MD data via parallel processing of the images. Large archives of film mammograms could then be rapidly revisited and read to answer epidemiologic research questions. Images from current and future studies may also be read at the same time as they are acquired, and the resultant readings, which could encompass both PD and additional mammographic features, could perhaps be used to estimate breast cancer risk better for each individual when incorporated into current breast cancer prediction tools, such as the Gail or the Claus models.

We acknowledge the weakness of using a largely postmenopausal study population, which, on average, has lower mammographic density than do premenopausal women. Caution is needed when evaluating mammograms with very high density values with the new method. In addition, the generalizability of the new automatic mammographic density thresholding method is currently limited to the MLO images (taken from an oblique or angled view) analyzed in this study. In many countries, the MLO view is preferred over lateral, perpendicular projections during routine screening mammography, as more of the breast tissue is visible in the upper outer quadrant of the breast and the axilla. Further work is required to extend the application of the method to other projections (for example, cranial-caudal, mediolateral). The high-throughput capacity of an automated method makes it feasible to base future assessments of MD on more than one view.

The generalizability of our new MD measurement method is at present confined to digitized screen film mammograms. With the paradigm transition from analogue to digital mammography, it is of high clinical relevance to extend the use of the automated PD thresholding method to digital mammograms. In contrast with current applications used to determine MD from digital mammograms, which have to be present as the image is being acquired by the machine, ImageJ can be applied at any time after image acquisition, making it feasible to read digital images retrospectively. However, many concerns must be addressed before MD can be confidently measured from processed digital mammograms in general. A more detailed discussion of this topic is beyond the scope of this article. Nevertheless, the availability of phenomenal archives of unread film mammograms for historical cohorts with good follow-up data justifies the development of an automatic tool.

Despite the remarkably strong influence of MD on breast cancer risk, it has had limited influence in clinical decision making and has not yet been included in any established risk-prediction tool. It is, however, likely that the purpose of future mammography screening programs will not be limited to the detection of early breast cancers, but also to stratify women according to their individual risk of breast cancer. Such stratification will make it possible to tailor screening intervals based on individual risk, add complementary diagnostic techniques (for example, ultrasound or magnetic resonance imaging), and select high-risk women for appropriate preventive interventions (for example, pharmacoprophylaxis). A robust, fully automated thresholding technique that can assess density in an objective and high-throughput manner is the first step to achieving these goals, with the ultimate aim of reducing the incidence of and mortality from breast cancer.

## Conclusions

We describe a novel method for using a public domain software for the automated analysis of mammographic density, with the intent of duplicating the findings of an established method (Cumulus), and improving the risk associations of mammographic density and breast cancer risk. Further work is required to validate and extend the application to mammographic images of other views and those produced by a digital mammography system.

## Abbreviations

AIC: Akaike information criterion; BMI: body mass index; CI: confidence interval; MD: mammographic density; MLO: mediolateral oblique; OR: odds ratio; *P: P *value; PC: principal component; PD: percentage density; *r*: Pearson product-moment correlation coefficient.

## Competing interests

The authors declare that they have no competing interests.

## Authors' contributions

PH, LS, KC, and KH conceived and designed the study. BH, AS, and LE collected and assembled data, and JL, KH, and LS were responsible for data analysis and interpretation. All authors contributed to manuscript writing and read and approved the final manuscript.

## Supplementary Material

Additional file 1 **Figure S1. An example of a digitized mammogram before and after thresholding and application of the watershed algorithm by using different global thresholding algorithms**. Click here for file

Additional file 2 **Figure S2. Scree plot showing the proportion of variance explained for from principal component analysis of 772 ImageJ variables**. PC, Principal component. Click here for file

Additional file 3 **Table S1**. **Weights of original variables**. Each principal component (PC) is a linear combination of the original variables. Loading values represent relative contributions of the original variables to each PC. "Total" measurements are made on particles of at least 5 pixels. Particles1, -2, and -3 denote measurements on particle size ranges of 5 to 100, 101 to 1,000, and at least 1,001 pixels, respectively. Click here for file

Additional file 4 **Table S2. Descriptive characteristics of study population by training or test subgroups**. Click here for file

Additional file 5 **Nonzero coefficients of penalized regression models**. Click here for file

## References

[B1] Boyd NF Lockwood GA Martin LJ Knight JA Byng JW Yaffe MJ Tritchler DL Mammographic densities and breast cancer risk Breast Dis 1998 14 113 126 15687568 10.3233/bd-1998-103-412

[B2] Boyd NF Dite GS Stone J Gunasekara A English DR McCredie MR Giles GG Tritchler D Chiarelli A Yaffe MJ Hopper JL Heritability of mammographic density, a risk factor for breast cancer N Engl J Med 2002 14 886 894 10.1056/NEJMoa013390 12239257

[B3] Boyd NF Guo H Martin LJ Sun L Stone J Fishell E Jong RA Hislop G Chiarelli A Minkin S Yaffe MJ Mammographic density and the risk and detection of breast cancer N Engl J Med 2007 14 227 236 10.1056/NEJMoa062790 17229950

[B4] Boyd NF Martin LJ Rommens JM Paterson AD Minkin S Yaffe MJ Stone J Hopper JL Mammographic density: a heritable risk factor for breast cancer Methods Mol Biol 2009 14 343 360 10.1007/978-1-60327-492-0_15 19107441

[B5] Boyd NF Rommens JM Vogt K Lee V Hopper JL Yaffe MJ Paterson AD Mammographic breast density as an intermediate phenotype for breast cancer Lancet Oncol 2005 14 798 808 10.1016/S1470-2045(05)70390-9 16198986

[B6] Wolfe JN Risk for breast cancer development determined by mammographic parenchymal pattern Cancer 1976 14 2486 2492 10.1002/1097-0142(197605)37:5<2486::AID-CNCR2820370542>3.0.CO;2-8 1260729

[B7] Gram IT Funkhouser E Tabar L The Tabar classification of mammographic parenchymal patterns Eur J Radiol 1997 14 131 136 10.1016/S0720-048X(96)01138-2 9097055

[B8] Byng JW Boyd NF Fishell E Jong RA Yaffe MJ The quantitative analysis of mammographic densities Phys Med Biol 1994 14 1629 1638 10.1088/0031-9155/39/10/008 15551535

[B9] ImageJ, U. S. National Institutes of Health, Bethesda, Maryland, USA http://imagej.nih.gov/ij/

[B10] Magnusson C Baron J Persson I Wolk A Bergstrom R Trichopoulos D Adami HO Body size in different periods of life and breast cancer risk in post-menopausal women Int J Cancer 1998 14 29 34 10.1002/(SICI)1097-0215(19980330)76:1<29::AID-IJC6>3.0.CO;2-# 9533758

[B11] Magnusson C Colditz G Rosner B Bergstrom R Persson I Association of family history and other risk factors with breast cancer risk (Sweden) Cancer Causes Control 1998 14 259 267 10.1023/A:1008817018942 9684706

[B12] Ludvigsson JF Otterblad-Olausson P Pettersson BU Ekbom A The Swedish personal identity number: possibilities and pitfalls in healthcare and medical research Eur J Epidemiol 2009 14 659 667 10.1007/s10654-009-9350-y 19504049 PMC2773709

[B13] Kittler J Illingworth J Minimum error thresholding Pattern Recogn 1986 14 41 47 10.1016/0031-3203(86)90030-0

[B14] Auto Threshold http://pacific.mpi-cbg.de/wiki/index.php/Auto_Threshold

[B15] Lindstrom S Vachon CM Li J Varghese J Thompson D Warren R Brown J Leyland J Audley T Wareham NJ Loos RJF Paterson AD Rommens J Waggott D Martin LJ Scott CG Pankratz VS Hankinson SE Hazra A Hunter DJ Hopper JL Southey MC Chanock SJ dos Santos-Silva I Liu J Eriksson L Couch FJ Stone J Apicella C Czene K Common variants in ZNF365 are associated with both mammographic density and breast cancer risk Nat Genet 2011 14 185 187 10.1038/ng.760 21278746 PMC3076615

[B16] Tibshirani R Regression shrinkage and selection via the LASSO J R Stat Soc B Methodol 1996 14 267 288

[B17] Tibshirani R The LASSO method for variable selection in the Cox model Stat Med 1997 14 385 395 10.1002/(SICI)1097-0258(19970228)16:4<385::AID-SIM380>3.0.CO;2-3 9044528

[B18] Goeman JJ L1 penalized estimation in the Cox proportional hazards model Biomed J 2010 14 70 84 10.1002/bimj.200900028 19937997

[B19] DeLong ER DeLong DM Clarke-Pearson DL Comparing the areas under two or more correlated receiver operating characteristic curves: a nonparametric approach Biometrics 1988 14 837 845 10.2307/2531595 3203132

[B20] R Development Core Team R: A Language and Environment for Statistical Computing 2011 Vienna, Austria: R Foundation for Statistical Computing

[B21] Akaike H BN Petrov, F Csaki Information theory and an extension of the maximum likelihood principle Second international symposium on information theory 1973 Budapest: Academiai-Kiado 267 281

[B22] Heine JJ Carston MJ Scott CG Brandt KR Wu FF Pankratz VS Sellers TA Vachon CM An automated approach for estimation of breast density Cancer Epidemiol Biomarkers Prev 2008 14 3090 3097 10.1158/1055-9965.EPI-08-0170 18990749 PMC2705972

[B23] Kallenberg MG Lokate M van Gils CH Karssemeijer N Automatic breast density segmentation: an integration of different approaches Phys Med Biol 2011 14 2715 2729 10.1088/0031-9155/56/9/005 21464531

[B24] Tice JA Cummings SR Smith-Bindman R Ichikawa L Barlow WE Kerlikowske K Using clinical factors and mammographic breast density to estimate breast cancer risk: development and validation of a new predictive model Ann Intern Med 2008 14 337 347 18316752 10.7326/0003-4819-148-5-200803040-00004 PMC2674327

[B25] Vacek PM Skelly JM Geller BM Breast cancer risk assessment in women aged 70 and older Breast Cancer Res Treat 2011 14 291 299 10.1007/s10549-011-1576-1 21604157

[B26] Amir E Evans DG Shenton A Lalloo F Moran A Boggis C Wilson M Howell A Evaluation of breast cancer risk assessment packages in the family history evaluation and screening programme J Med Genet 2003 14 807 814 10.1136/jmg.40.11.807 14627668 PMC1735317

[B27] Boyd NF Martin LJ Yaffe MJ Minkin S Mammographic density and breast cancer risk: current understanding and future prospects Breast Cancer Res 2011 14 223 10.1186/bcr2942 22114898 PMC3326547

[B28] Nielsen M Karemore G Loog M Raundahl J Karssemeijer N Otten JD Karsdal MA Vachon CM Christiansen C A novel and automatic mammographic texture resemblance marker is an independent risk factor for breast cancer Cancer Epidemiol 2011 14 381 387 10.1016/j.canep.2010.10.011 21146484

