# Supplementary figures and images for: High-throughput mammographic-density measurement: a tool for risk prediction of breast cancer
Source: Breast Cancer Res. 2012 Jul 30;14(4):R114. doi: 10.1186/bcr3238 (PMC3680940; doi:10.1186/bcr3238)

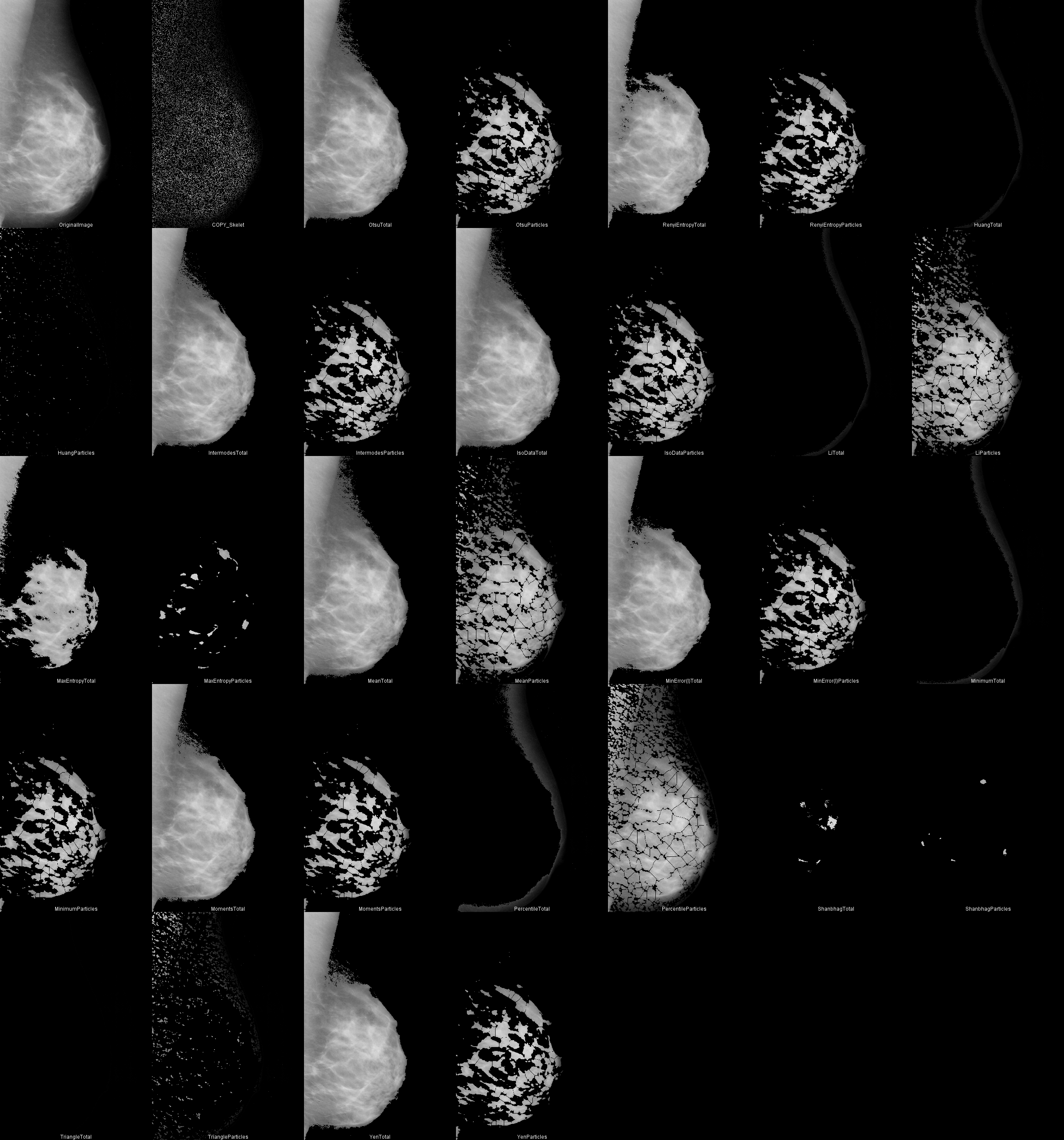

Supplement: Additional file 1 — Figure S1. An example of a digitized mammogram before and after thresholding and application of the watershed algorithm by using different global thresholding algorithms. [file bcr3238-S1.ZIP › xC711835219950502RMLO16010.tif]

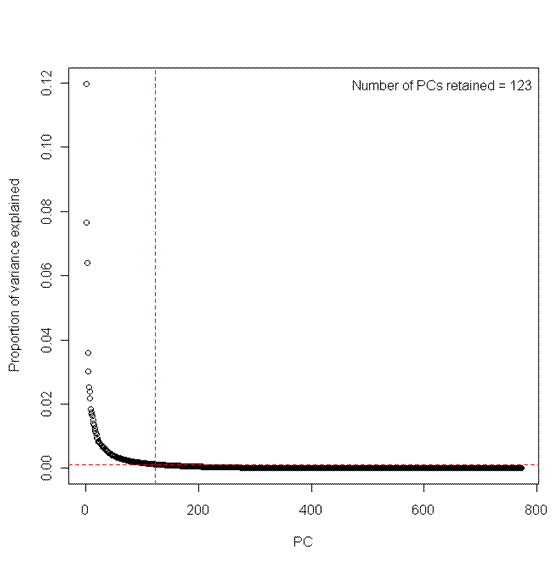

Supplement: Additional file 2 — Figure S2. Scree plot showing the proportion of variance explained for from principal component analysis of 772 ImageJ variables. PC, Principal component. [file bcr3238-S2.GIF]
