# Supplementary material for: High-throughput mammographic-density measurement: a tool for risk prediction of breast cancer
Source: Breast Cancer Res. 2012 Jul 30;14(4):R114. doi: 10.1186/bcr3238 (PMC3680940; doi:10.1186/bcr3238)
Supplement: Additional file 4 — Table S2. Descriptive characteristics of study population by training or test subgroups. [file bcr3238-S4.DOCX]

Table S2. Summary characteristics of study population by training or test subgroups.

| **Characteristic** | **Training set**  (733 breast cancer cases and 748 controls) | | **Test set**  (765 breast cancer cases and 747 controls) | | **P** |
| --- | --- | --- | --- | --- | --- |
| **Median** |  |  |  |  |  |
| Percent density (%) | 13.5 |  | 14.3 |  | 0.228 |
| Absolute dense area (cm^2^) | 19.9 |  | 20.8 |  | 0.443 |
|  |  |  |  |  |  |
| **Mean (SD)** |  |  |  |  |  |
| Age at diagnosis or reference date (y) | 62.1 (7.0) |  | 61.9 (7.1) |  | 0.375 |
| Age at mammogram (y) | 61.8 (7.0) |  | 61.6 (7.1) |  | 0.359 |
| Age at menarche (y) | 13.6 (1.4) |  | 13.5 (1.4) |  | 0.421 |
| Age at menopause (y) | 50.3 (3.9) |  | 50.3 (3.7) |  | 0.781 |
| BMI at diagnosis or reference date (kg/m^2^) | 25.2 (3.7) |  | 25.1 (3.7) |  | 0.839 |
| Alcohol consumption (g/day) | 2.3 (4.2) |  | 2.7 (5.0) |  | 0.235 |
| Percent density (%) | 17.9 (15.2) |  | 18.5 (15.8) |  | 0.454 |
| Absolute dense area (cm^2^) | 26.4 (22.6) |  | 27.1 (23.1) |  | 0.441 |
|  |  |  |  |  |  |
| **Frequency, number (%)** |  |  |  |  |  |
| Categorical percent density (%) |  |  |  |  | 0.570 |
| <10 | 576 (38.9) |  | 581 (38.4) |  |  |
| 10-24 | 531 (35.9) |  | 513 (33.9) |  |  |
| 25-49 | 301 (20.3) |  | 333 (22.0) |  |  |
| 50-74 | 70 (4.7) |  | 83 (5.5) |  |  |
| ≥75 | 3 (0.2) |  | 2 (0.1) |  |  |
| Categorical absolute dense area (cm^2^) |  |  |  |  | 0.213 |
| <10 | 367 (24.8) |  | 396 (26.2) |  |  |
| 10-24 | 513 (34.6) |  | 467 (30.9) |  |  |
| 25-49 | 388 (26.2) |  | 419 (27.7) |  |  |
| 50-74 | 153 (10.3) |  | 172 (11.4) |  |  |
| 75-99 | 47 (3.2) |  | 39 (2.6) |  |  |
| ≥100 | 13 (0.9) |  | 19 (1.3) |  |  |
| Parity and age at first birth |  |  |  |  | 0.939 |
| Nulliparous | 165 (11.1) |  | 173 (11.4) |  |  |
| 1-3 children, age at first birth <25y | 550 (37.1) |  | 561 (37.1) |  |  |
| 1-3 children, age at first birth 25-29y | 408 (27.5) |  | 411 (27.2) |  |  |
| 1-3 children, age at first birth ≥30y | 208 (14.0) |  | 227 (15.0) |  |  |
| ≥4 children, age at first birth <25y | 30 (2.0) |  | 30 (2.0) |  |  |
| ≥4 children, age at first birth ≥25y | 119 (8.0) |  | 109 (7.2) |  |  |
| Hormone replacement therapy |  |  |  |  | 0.074 |
| Never used hormones | 806 (54.4) |  | 760 (50.3) |  |  |
| Ever used hormones | 673 (45.4) |  | 750 (49.6) |  |  |
| Unknown status of hormone use | 2 (0.1) |  | 2 (0.1) |  |  |
| Family history of breast cancer (Ever) | 155 (10.5) |  | 171 (11.3) |  | 0.603 |
| Benign breast disease (Ever) | 163 (11.0) |  | 188 (12.4) |  | 0.247 |
